# Endothelial Progenitor Cells: New Targets for Therapeutics for Inflammatory Conditions With High Cardiovascular Risk

**DOI:** 10.3389/fmed.2018.00200

**Published:** 2018-07-10

**Authors:** Nicola Edwards, Alexander W. W. Langford-Smith, Fiona L. Wilkinson, M. Yvonne Alexander

**Affiliations:** ^1^Cardiovascular Science, Centre for Bioscience, Faculty of Science and Engineering, Manchester Metropolitan University, Manchester, United Kingdom; ^2^Manchester Academic Health Science Centre, Manchester, United Kingdom

**Keywords:** endothelial progenitor cells, glycocalyx, cardiovascular, mitochondrial function, inflammation, lupus, rheumatoid arthritis, diabetes

## Abstract

Over the past decade, we have witnessed an exponential growth of interest into the role of endothelial progenitor cells (EPCs) in cardiovascular disease. While the major thinking revolves around EPC angiogenic repair properties, we have used a hypothesis-driven approach to discover disease-related defects in their characteristics and based on these findings, have identified opportunities for functional enhancement, which offer an exciting avenue for translation into clinical intervention. In this review, we focus on two groups; circulating myeloid angiogenic cells (MACs) and late outgrowth endothelial colony forming cells (ECFCs), and will discuss the unique properties and defects of each population, as new insights have been gained into the potential function of each sub-type using current techniques and multiomic technology. We will discuss their role in inflammatory disorders and alterations in mitochondrial function. In addition, we share key insights into the glycocalyx, and propose this network of membrane-bound proteoglycans and glycoproteins, covering the endothelium warrants further investigation in order to clarify its significance in ECFC regulation of vascularization and angiogenesis and ultimately for potential translational therapeutic aspects.

## Endothelial damage and repair in high-risk disease conditions

### Endothelial damage

Endothelial dysfunction is a preceding factor in the development of cardiovascular disease, with vascular damage leading to atherogenesis, and plaque formation (1). Endothelial damage may be mediated by a number of biological stimuli, such as inflammatory mediators, and hypoxia (2). Indeed, many conditions characterized by inflammation, of either a chronic or transient nature, are associated with a high risk of endothelial dysfunction. These “high risk” conditions include autoimmune diseases such as systemic lupus erythematosus (SLE) (3) and rheumatoid arthritis (RA) (4), as well as chronic conditions such as type 2 diabetes mellitus (T2DM) (5). The association between cardiovascular and autoimmune disease has been recognized for a number of years (6), where it is known that cardiovascular risk in young women with SLE is increased 50 fold (7, 8). These patients experience endothelial dysfunction prior to accelerated subclinical atherosclerosis, possibly due to the sustained activation of the immune response, including production of pro-atherogenic hormones and immune complexes (9), and research has shown that endothelial progenitor cells (EPCs) are likely to play a role in this process (10). In addition, T2DM patients likewise experience endothelial dysfunction due to the accumulation of advanced glycated end products, oxidized low density lipoprotein (oxLDL), and oxidative stress, making the diabetic patient prone to atherogenesis, and increased cardiovascular risk (5), with recent evidence suggesting this could be influenced by a decrease in the number and function of EPCs (11).

### Endothelial homeostasis and repair

EPCs are important for vascular homeostasis and repair, where differences in their number and function in health and disease are apparent (12). It is well recognized that during stress or endothelial activation, EPCs can be mobilized, with their numbers being increased in the circulation (13). Growing evidence suggests that EPCs could be a link between a defective homeostatic or endogenous repair mechanism and vascular dysfunction (14) and in this regard could have a future impact as a biomarker of atherosclerosis and vascular disease (15). Characteristics of the late outgrowth endothelial colony forming cell (ECFC) phenotype, including cell-surface markers necessary for adhesion to the vascular endothelium, and their angiogenic capacity, support the suggestion that ECFCs are a key sub-population of EPCs involved in vascular repair (16, 17). Tissue injury studies in animal models have demonstrated mobilization and migration of EPCs from the bone marrow niche, followed by homing to the site of vascular damage, where they modulate repair through angiogenesis, neovascularization, and endothelial cell replenishment, all of which has been elegantly reviewed elsewhere (18, 19). Although the evidence for the role of EPCs in atherosclerosis has met with some discrepancy, it is likely due to differences in the particular EPC phenotype investigated, their distinctive functional effects, as well as initiating factors triggering their action. A recent report used EPCs, defined by their combined expression of CD34^+^, CD133^+^, and KDR^+^, and demonstrated an association between a high EPC count with less coronary plaque burden of a stented vessel segment, which adds to previous findings of their protective role in atherosclerosis (20). In addition, it has been reported that factors released from atherosclerotic plaques *ex vivo*, induce, not only mobilization of EPCs, but also EPC expression of angiogenic factors (21).

In parallel, there is evidence that MACs are also of utmost importance in their contribution to angiogenesis, tissue regeneration and endothelial repair (22, 23), where these precursor cells exert paracrine and trophic effects that influence the host microenvironment (24, 25). The following sections will describe mechanisms underpinning vascular dysfunction in inflammatory conditions with high risk of cardiovascular disease (CVD) and consider potential therapeutic options aimed at improving progenitor cell reparative function (26, 27) as a novel approach to exploit endogenous repair processes.

## Defective EPCs in aging and diabetes

### Aging

It would appear that numbers of circulating ECFCs reduce over time, such that older volunteers are found to have fewer ECFCs than their younger counterparts (28, 29). This is mirrored in coronary heart disease, reinforcing the connections between ECFC dysfunction, aging, and cardiovascular risk (28). However, there are also reports that bone-marrow derived progenitor cell numbers remain stable, suggesting that, in some cases, the decline in reparatory ability may be due to cellular impairments, in their homing, angiogenic capacity or their propensity for senescence and premature cell death (28, 30, 31). Studies have shown that late ECFCs from elderly volunteers demonstrate impaired migration, proliferation and adhesion properties compared to those from young participants (29, 32, 33), and show a reduced capacity for re-endothelialization and incorporation into a damaged vasculature (29).

ECFCs isolated from older individuals develop a decline in their response to signaling pathways. Among the many mechanisms that may underpin the impaired function of EPCs in disease, our own studies have demonstrated a reduction in 6-O-sulphation of heparan sulfate in aging ECFCs, suggesting the glycocalyx may play a role in the aging decline of vascular health (33). In addition, Kushner et al. report that ECFCs from older subjects, compared to their younger counterparts, have an increased sensitivity to apoptotic stimuli and demonstrate an increased level of intracellular caspase-3, along with accelerated senescence, which was linked to a loss of telomerase, and a pro-thrombotic phenotype (34). Xia et al. have shown altered CXCR4/JAK signaling in the elderly is linked to a reduced capacity for ECFC homing and re-endothelialization (29), which may concordantly induce anti-atherosclerotic EPC activity and up regulate expression of vascular endothelial growth factor (VEGF) receptors, as discussed elsewhere (35). Interestingly, Heiss et al. found increased concentrations of VEGF in the blood of older individuals although ECFC responses to the protein were muted (32, 36, 37), thus implying an increased effort to mobilize ECFCs and effect vascular repair (32).

Studies have been carried out with MACs also, where young patients with type 1 diabetes mellitus have shown significantly higher levels of MACs compared to adult patients, and where a direct correlation was found between MAC number and disease duration, when greater than 10 years (38). The authors propose that the high levels of MACs in the young patients might protect vessels against endothelial dysfunction and damage and such protection would be less effective in older subjects, who had lower EPC numbers (38). In addition, older MACs were shown to be more susceptible to oxidative stress due to reduced activity of antioxidant proteins such as GPX1, thus rendering them vulnerable to apoptosis (38, 39).

### Diabetes

As with aging, T2DM is associated with a reduction in circulating ECFCs, and also shows an impaired VEGF-driven mobility (38, 40–42), as well as major deficits in vital functions such as differentiation and proliferation. The effects of a hyperglycaemic environment on ECFC number and function are comprehensively reviewed by Kang et al. (43). Furthermore, the reduced numbers of ECFCs in T2DM have been associated with poor glycaemic control, and increased arterial stiffness (41). Hyperglycaemia may also enable uncoupling of intracellular eNOS, rendering ECFCs susceptible to ROS and further migratory incapability (42), although of note, function can be restored following improved glycaemic control (40), indicating the potential for lifestyle and therapeutic options to improve vascular repair. This is particularly important in terms of peripheral vascular disease and poor wound healing, which often results in diabetic foot ulceration and amputation (44). The severely diminished ECFC number and function, which is apparent in T2DM, also correlates to the prevalence of atherosclerosis in the lower limbs (44, 45). In this case, the ECFCs demonstrate impaired clonogenicity and adhesion (45), which, when coupled with impaired homing, may contribute to the delayed wound healing observed in diabetes. As an added complication in a diabetic microenvironment, ECFCs appear to concurrently undergo a pro-calcific shift, expressing osteocalcin, and bone alkaline phosphatase, thus promoting the drive toward vascular calcification, which is so prevalent in diabetic vasculopathy (46). This phenomenon renders ECFCs not only important in endothelial dysfunction, but also in smooth muscle cell osteogenic differentiation.

## Mechanisms of EPC dysfunction in aging and diabetes

### EPCs and the glycocalyx

Previous research by our group supports the theory of a decline in function with age, where we demonstrated structural changes in heparan sulfate within the glycocalyx of aged ECFCs, compared to those isolated from younger volunteers and cord blood. Our findings also demonstrate an association with reduced sensitivity to VEGF (33). Since heparan sulfate is indeed a ligand for VEGF, we suggest that aged ECFCs may be less sensitive to damage signals through reduced protective/reparative ligand-binding (33, 38, 47). Impairments in syndecan 4, another member of the heparan sulfate proteoglycan receptor family and involved in SDF-induced cell migration, has been shown to contribute to impaired ECFC function. The extracellular domain of syndecan 4 is shed from the cell surface of ECFCs in response to ROS-induced accumulation of advanced glycation end products, leading to impaired migration of the syndecan4 deficient ECFCs (48).

In other cell types, the glycocalyx also has a role in immune regulation, mediated by the binding of complement factor H to specific, age-related alterations in the sulphation patterning of heparan sulfate (49, 50). Furthermore, these age-related changes that we have identified in the glycocalyx of ECFCs (33, 49) could be caused by the accumulation of metal ions, including cadmium within the matrix, resulting in ROS-mediated damage to the glycocalyx, and mitochondrial dysfunction (51), however links between metal ions and ECFC regulation remain to be further investigated.

### EPCs and mitochondria

EPCs have previously been demonstrated to form cell-to-cell connections and, via tunneling nanotubes (TNT), transfer mitochondria and other organelles to endothelial cells. This TNT mitochondrial transfer can rescue senescent endothelial cells and change cell fate (52, 53). Further details of TNT mitochondrial trafficking in health and disease can be found in other reviews (54, 55). The energy required for the normal function of most endothelial cell phenotypes is primarily by glycolysis, however, the energy requirements for repair and angiogenesis are considerable and thus require the activation and proliferation of mitochondria (56, 57). In light of this role in angiogenesis, mitochondria are key integrators of environmental and disease signals (58), and they are crucial to the mechanism underpinning many other factors that influence EPC behavior discussed in this review. For example, among the many cellular processes which influences mitochondrial function and metabolic homeostasis are different shear stress conditions (58), in both mediating and also causing inflammatory responses (59, 60) and in diabetes (61). Of note, we have recently identified impaired angiogenic function and altered mitochondrial activity in ECFCs isolated from patients with diabetes and foot ulcers (62).

There is also a dynamic interplay between mitochondrial function, the glycocalyx and extracellular cell matrix; for example, the glycocalyx can be damaged/changed by ROS produced in the mitochondria (63) in certain conditions, while the stiffness of the matrix can also affect mitochondrial function in other cases (43, 64). Although many of these mechanisms have not been demonstrated in EPCs it is likely they also have a role, however, further work is warranted to enhance our understanding of the complex interplay between EPC function, the glycocalyx/matrix, mitochondria, and other disease and aging stimuli.

### EPCs and shear stress

CD31, or platelet endothelial cell adhesion molecule-1 (PECAM-1), is a 140kDa type I integral membrane glycoprotein often used as a marker of EPCs as well as the more mature endothelial cells and is known to play various roles in vascular biology, including angiogenesis, platelet function, and thrombosis. It is also a mechanosensor of the endothelial cell response to fluid shear stress. It is thought that ECFC filopodial processes may play a role in cellular communication, and regulating cell to cell contact by allowing a sensory response to circulatory or sheer stress. Further work is required to gain insight into the effects of sheer stress on MAC and ECFC function. Enhanced signaling and re-endothelialization has been shown to be restored in elderly ECFCs, following shear stress treatment (29). However, there is little understanding of the influence of flow stress changes within the glycocalyx and extracellular matrix and how this might influence MAC or ECFC behavior, providing the impetus for further studies into novel mechanobiological studies of how these cells respond to changes in physiological or turbulent flow.

## Progenitor cell impairments in inflammatory rheumatic diseases

### Rheumatoid arthritis

Reports pertaining to progenitor cell numbers in autoimmune rheumatic conditions are conflicting due to the different methods of progenitor cell characterization and patient inclusion criteria used by various groups. While some studies suggest a decrease in CD34^+^ cells in rheumatoid arthritis (RA) (65), other studies demonstrate increased levels (66), or indeed no change at all in number (23). Although reports of EPC number in RA are inconsistent, low levels of CD34^+^/KDR^+^ cells have been associated with carotid atherosclerosis in patients (67), suggesting that a reduction in number may be more representative of vascular dysfunction than inflammatory activity (68). Furthermore, it has been suggested that ECFC depletion is associated with disease progression, as patients experiencing long-term disease appear to show a decline in ECFC numbers, regardless of age, compared to those with recent disease onset, whose ECFC numbers match those of healthy participants (69). Although discrepancies exist in respective RA studies because of differences in MAC and ECFC isolation methods (70), including their seeding density, the matrix used for coating culture dishes, the markers in use for characterization, and the potential variances in drug regimens of study participants before isolation of their cells, it is clear that both MACs and ECFCs do have potential to act as targets for therapeutic improvement in disease (68).

### Systemic lupus erythematosus

Patients with systemic lupus erythematosus (SLE) have an elevated vascular risk due to an early onset of atherosclerosis, which appears to be independent of traditional CVD risk factors and associated with an altered interferon-α (IFNα) signaling pathway. It has been shown that IFNα alters the balance between endothelial cell apoptosis and vascular repair which is governed by both ECFCs and MACs (71, 72). When focusing on CD34^+^ cells, it becomes clear that the majority of studies find decreased levels of circulating MACs in SLE patients (68). A reduction in numbers of CD34^+^/KDR^+^ MACs in SLE patients, has been attributed to increased apoptosis, which is also reported in patients with stable disease in remission, supporting the proposal of chronically decreased levels throughout the disease, rather than solely during a disease flare (73). Moonen et al. described MACs with unusual morphology (74), while Denny et al. found decreased ability to express pro-angiogenic cytokines such as VEGF (75), which they correlated with impaired VEGF-driven migration (76), and was supported by a subsequent study in our group by Williamson et al. (33). SLE ECFCs have also been shown to have fundamental impairments in critical functions such as colony forming ability and proliferation (76), as well as reduced migration and tube forming capabilities (75). These findings are strengthened by Deng et al. who found that while ECFCs isolated from patients with SLE are highly activated and have elevated expression of interleukin-6 (IL-6) and intracellular adhesion molecule-1 (ICAM-1) compared to control subjects, they are impaired in their basic physiological function (77).

## Inflammatory signaling and restoration of ECFC function

### Cytokine-induced endothelial damage

The inflammatory environment plays a vital role in ECFC function and maturation; IFNα is most often associated with SLE but may also be elevated in RA and demonstrates striking correlations with ECFC number and function, suggesting a role in the induction of differentiation of the ECFC population (23). One theory states that IFNα drives premature differentiation of ECFCs to a more mature phenotype, with little reparatory potential, therefore, even if the cells are found at healthy levels, their ability to repair vascular damage is severely limited (69). Impairments in ECFC maturation and function are likewise linked to IFN signaling in a type I IFN receptor knockout murine model of SLE, where Thacker et al. demonstrated increased ECFC number and function, with improved neoangiogenesis and differentiation (78). It was suggested that type 1 IFN receptor activation causes the impairment by transcriptional repression of IL-1β, upregulation of inflammasome components, such as caspase-1 and a skew toward pro-inflammatory IL-18. Indeed, blockade of both caspase-1 and IL-18 enhance differentiation of progenitor cells (79). Denny et al. support the damaging effects caused by an altered IFNα signature in an *in vitro* SLE model, where they demonstrate increased production of IFNα by both MACs and ECFCs, which become cytotoxic to the cells, supporting apoptosis and preventing growth of a confluent monolayer (75). Administration of IFNα was shown to enhance thrombosis and platelet activation in a lupus-prone mouse model (78) and high IFNα levels have been suggested as an independent risk factor for cardiovascular disease in both SLE and RA (69, 80). In addition, IL-18 has been associated with vascular stiffness and plaque instability, acting as an independent predictor of cardiovascular mortality in patients with subclinical atherosclerosis (79).

A further hallmark of inflammation is elevated expression of systemic or tissue tumor necrosis factor α (TNFα), which is another key cytokine elevated in autoimmune rheumatic disease (81); accordingly, treatment of harvested healthy ECFCs with TNFα has been shown to impair proliferation, migration and tube formation in these cells, and increase apoptosis *in vitro* (82). As with increased levels of IFNα contributing to ECFC dysfunction, so the increased levels of TNFα and subsequent damage to MACs and ECFCs, may contribute to a poor vascular repair in these patients. Additional members of the TNF family may assert detrimental effects on an altered differentiation programme of progenitor cells. For example, osteoprotegerin (OPG), which inhibits osteoclastogenesis and is a marker of vascular calcification (83), has been shown to be inversely correlated with ECFC numbers in SLE patients, and linked to an increased rate of OPG-stimulated apoptosis compared to those from healthy participants, suggesting that the apoptotic cells could act as a nidus for calcified matrix progression. The same study demonstrated that ECFCs increased basal production of ROS, suggesting that the increased inflammation and exposure to apoptotic stressors associated with SLE increased the likelihood of both ECFCs and MACs becoming exhausted and succumbing to apoptosis (84).

### miRs and microvesicles: their effects on EPCs

MicroRNAs (miRs) are critical players in posttranscriptional regulation of almost all genes influencing cellular processes, cell fate decisions, regulating epigenetic changes and contributing to the disease process, details of which are outside the scope of this review, but are elegantly reviewed elsewhere (85–87). Elucidation of the regulatory mechanisms controlled by miRs is an important step toward development of a novel therapy for cardiovascular disease and the co-morbidities associated with it. A study by Khoo et al. describes how differential expression of miR-193a-3p by ECFCs reduces proliferation, migration and tube forming ability by interacting with novel targets such as high mobility group box-1 (88). A further consideration is the reversal of this pathway, in which microvesicles (MVs) and exosomes derived from EPCs act upon the endothelium (89); indeed, circulating ECFC-MVs have been found to stimulate a pro-angiogenic effect upon endothelial cells, which is mediated by the transfer of mRNA carried within the MVs (90). Ranghino et al. expanded on this by establishing a connection between specific miRNA, such as miR-126, and neoangiogenesis, through the use of ECFC-MVs for resolution of hind limb ischaemia (91).

Endothelial microvesicles (EMVs) are membrane-bound, cellular-derived vesicles that exert paracrine or endocrine effects through the intercellular transfer of contents such as lipids, proteins, mRNA and microRNA (miRNA), and are thus intricately linked to endothelial dysfunction (92). Elevation in EMV levels is associated with coronary artery disease (93), plaque instability (94), cardiovascular risk (95, 96), and is also apparent in autoimmune rheumatic diseases, such as SLE, where EMVs are also associated with vascular dysfunction and poor disease control (97, 98). A small number of studies have demonstrated how EMVs produced by endothelial cells following induced inflammation are able to induce functional defects in EPCs, such as impaired angiogenesis (99, 100). miRNAs may also be present in EMVs released from activated cells compared to those from untreated cells, and could be involved in eliciting these effects (99).

### Epigenetic influences on EPC behavior

ECFCs isolated and expanded in culture maintain a phenotype related to the age, environment and pathologies of the individual donor; these epigenetic changes make these cells invaluable in the understanding the ECFC functionality in different conditions. A more detailed discussion of the histone modification and miR mechanism behind the epigenetic regulation in diabetes and other diseases can be found in other reviews (101, 102). It has also been identified that even ECFCs isolated from cord blood are epigenetically limited in their repair potential; Fraineau et al. recently identified a balance between histone modifications that increase gene expression (histone H3 lysine 4 trimethylation; H3K4me3) and those that inhibit it (histone H3 lysine 27 trimethylation; H3K27me3). Utilizing an inhibitor of the methyltransferase EZH2, that establishes the repressive H3K27me3 marks, Fraineau et al. demonstrated an increase in the expression of multiple pro-angiogenic pathways and an increase in vasculogenesis and blood-flow recovery in a hindlimb ischemia mouse model (103). Previously, a less targeted inhibition of histone deacetylases by Trichostatin A has also been shown to improve vasculogenesis in the hindlimb ischemia mouse model (104). Therefore, the pharmacological targeting of epigenetic modifications could be a promising strategy to improve the repair capacity of ECFCs *ex vivo* before transplantation (11).

### Future mechanistic clinical consideration

In light of the defective progenitor cell function in the presence of an inflammatory environment described above, one could hypothesize that anti-inflammatory treatment might improve EPC number and function. However, progenitor cell impairments appear to be exacerbated by immunomodulatory treatments such as methotrexate and rapamycin, which have been shown to increase ECFC apoptosis *in vitro* (65, 105). These observations suggest that one of the clinical effects of anti-inflammatory treatment in humans may target the protective properties of MACS or ECFCs. This has particular relevance in SLE and RA patients treated with chronic high-dose immunosuppressants to counteract autoimmune disease flares (106). Therefore, it is critical to consider the long-term side-effects of these anti-inflammatory medications on vascular repair and the cells responsible for it and provides the impetus to study the effects of anti-inflammatory treatment on these reparative cells *in vivo*.

## Therapeutic strategies for improved ECFC function

### Anti-inflammatory agents

Improved understanding of progenitor cell subsets and the mechanistic problems surrounding disease-specific defects will enable development of targeted therapies to improve a patient's natural population of reparatory cells. Some current available therapies have potential for recovery of progenitor cell function; glucocorticoids and TNF-blocking treatments appear to boost progenitor cell numbers in RA patients, while antimalarial drugs, often prescribed to SLE patients, may also increase levels of ECFCs (23, 107). A number of monoclonal antibody therapies targeting cytokines have been approved for use in autoimmune conditions, including both anti-TNFα and anti-IFNα (108, 109), although little research has investigated their direct effects on progenitor cell function, and may be an area worthy of further study.

### Anti-hypertensive agents

Prostanoids may be another potential therapeutic target agent relevant to EPFC or MAC function; Iloprost, a prostacyclin analog and vasodilator, has been shown to increase ECFC numbers in systemic sclerosis, another autoimmune connective tissue disease. Following Iloprost infusion, cells demonstrate enhanced inhibitory regulation of apoptotic genes and increased VEGF expression, facilitating improved mobilization (110). This may be supported by increased presentation of adhesion factors by the endothelium, alongside increased release of ECFCs from the bone marrow, as described by Coppolino et al. (111) using a population of uraemic patients undergoing revascularization for peripheral limb ischaemia. Indeed, prostanoids such as Iloprost, have been proposed to improve ulcer healing and reduce the need for major amputation (112), although further work is required to link this to MAC or ECFC function.

### Glycomimetics as novel small molecule drugs

A promising approach is to target the action of proteoglycans such as heparan sulfate, which is present on the surface of ECFCs, and plays a vital role in processes including angiogenesis and wound healing through its varied sulphation patterning, regulating interactions with growth factors such as VEGF, as described above (33, 113). The synthesis of small molecule glycomimetic compounds removes the complexity of larger carbohydrates, enabling the study of such glycosaminoglycans (114) and their effect on cell fate and function. Of note, we have previously discovered a group of glycomimetics, which restore NO production and antioxidant activity in an *in vitro* model of lipid-induced endothelial dysfunction (113), and our preliminary data suggest the same glycomimetic compounds improve the function of ECFCs (62). Another mimetic, used by Chevalier et al. was shown to improve colony formation, proliferation and migration of ECFCs (115). Furthermore, Tong et al. developed a glycomimetic that accelerated wound healing and angiogenesis in a murine model of diabetes, demonstrating promising future options using glycomimetics to improve endogenous ECFC or MAC function for vascular repair, particularly in T2DM foot ulceration.

### Vitamin D supplementation

Natural solutions for progenitor cell therapy have also been considered. We have previously found that supplementation of SLE MACs with calcitriol, a vitamin D supplement, restores cell surface markers and angiogenicity, via reduced expression of CXCL10 (25). This supports other research, stating improvements in angiogenesis, proliferation and VEGF expression following vitamin D treatment (116). This is particularly relevant as reduced levels of vitamin D have been associated with low ECFC numbers, carotid intima-media thickness and arterial stiffening in rheumatoid arthritis (117). Vitamin D deficiency also results in impaired ECFC angiogenic capacity and interferon-stimulated genes in a murine model of SLE (118). In addition to deficiency, reduced ECFC expression of the vitamin D receptor has been linked to coronary artery disease; high glucose conditions also reduced vitamin D receptor expression in an *in vitro* diabetic cellular model (119). Moreover, calcitriol supplementation supported ECFC viability and colony forming ability in patients with T2DM (120), which could be exploited further as a potentially simple and cost-effective mode of enhancing the health of patients.

## Conclusion and future approaches

**Figure 1:**
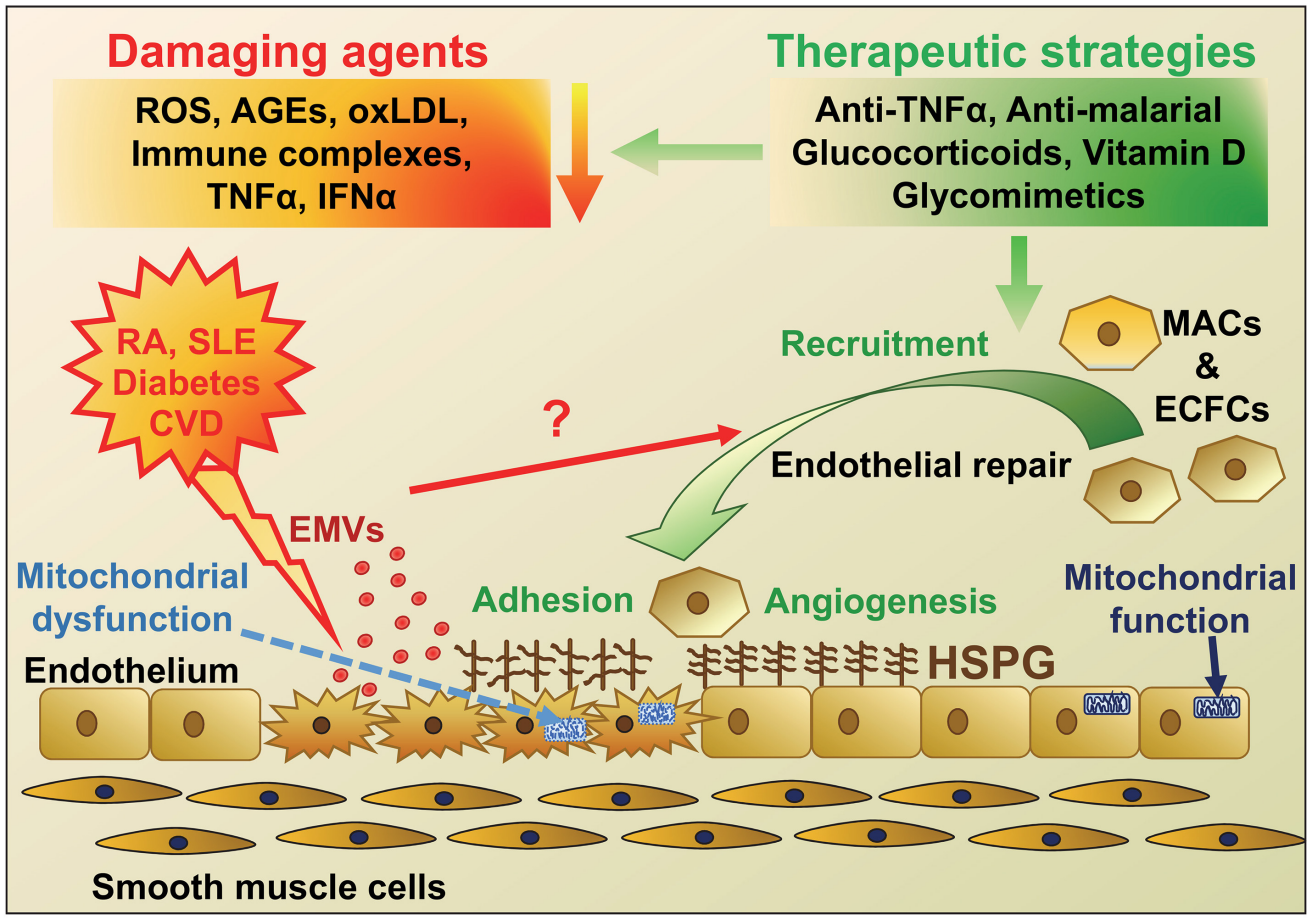
Schematic diagram highlighting the agents that cause endothelial damage in diseases with a high risk of developing cardiovascular disease (CVD) and potential therapeutic strategies for endothelial repair. The main protagonists of endothelial damage in diseases including rheumatoid arthritis (RA), Systemic Lupus Erythematosus (SLE), diabetes and CVD are reactive oxygen species (ROS), immune complexes, advanced glycation end products (AGEs), oxidized LDL, tumor necrosis factor-α (TNFα), and interferon-α (IFNα). Endothelial damage and activation leads to an increase in adhesion molecules and inflammatory cell infiltration, mitochondrial damage, as well as release of endothelial microvesicles (EMVs), which may instigate recruitment of endothelial progenitor cells (EPCs; myeloid angiogenic cells [MACs] and endothelial colony forming cells [ECFCs]) for endothelial repair. These disease conditions, along with aging, are also thought to change heparan sulfate proteoglycan (HSPG) structure on the cell surface, resulting in altered cell signaling and adhesion of EPCs and defective repair. Various therapeutic strategies could be employed to reduce the initial damage but also novel approaches using mimics of HSPG to target and improve HSPG signaling and repair are under investigation.

Regenerative medicine is now becoming a realistic innovative treatment strategy that could be applied to a range of chronic inflammatory disorders. Validation of the regenerative potential of adult MACs and ECFCs will be a prerequisite step before application of cell therapy in the clinical setting and although still an embryonic field, this challenge holds great promise for the future. Growing evidence demonstrates that both MACs and ECFCs play a key role in vascular homeostasis and the repair of endothelial damage, which has been summarized schematically in Figure 1. There has been a rapid rise in the number of publications in EPC function, where proliferation, migration, differentiation, apoptosis, and angiogenic tube formation have been studied. More recent research is focused on signaling pathways involved in these cellular processes. The “omic” technologies have been used in combination with bioinformatic analyses to identify transcriptional switches, miR involvement and their potential targets in both MACs and ECFCs, which contributes to their compromised function in disease.

How to best exploit the properties of EPCs to prevent the downstream effects of endothelial damage in a disease setting is a key area that warrants further investigation. Several approaches are being interrogated for the exploitation and application of MAC or ECFC cellular therapy, such as the use of nanoparticles as carriers for a controlled release of EPC secretome (121); the use of hydrogels for delivery of EPCs into ischemic tissue, which has been shown to increase therapeutic efficiency and efficacy of repair in animal models (122) and also encapsulation of drugs or growth factors for slow release to enhance the differentiation of progenitor cells *in vivo*. Animal models are being used to understand signaling pathways involved in vessel repair and ways to increase endogenous EPC number and function. A few specific pharmacological strategies are being investigated to improve their vasculogenic properties before being re-administrated. However, at the moment the focus is toward myocardial ischemia and peripheral vascular disease, but has potential for a much broader range of diseases in the future. Questions remain to be answered over the use of MACs and ECFC for cell therapy in terms of their isolation, culture, survival, function, regulation and the timing and mode of administration into the tissue. Despite this, the concept of EPCs as a new therapeutic, or as part of the armamentarium for regenerative medicine is a new, dynamic area of research that will bring further insight in the future.

## Author contributions

All authors listed have made a substantial, direct and intellectual contribution to the work, and approved it for publication.

### Conflict of interest statement

The authors declare that the research was conducted in the absence of any commercial or financial relationships that could be construed as a potential conflict of interest.
